# Inhibiting CB1 receptors improves lipogenesis in an *in vitro* non-alcoholic fatty liver disease model

**DOI:** 10.1186/1476-511X-13-173

**Published:** 2014-11-18

**Authors:** Dongmei Shi, Xi zhan, Xiaofeng Yu, Minglei Jia, Ying Zhang, Jianfeng Yao, Xiaona Hu, Zhijun Bao

**Affiliations:** Department of Gastroenterology, Huadong Hospital, Medical School of Shanghai Fudan University, Shanghai, 200040 China; Department of General Surgery, Ruijin Hospital, Medical School of Shanghai Jiaotong University, Shanghai Institute of Digestive Surgery, Shanghai, 200025 China

**Keywords:** Endocannabinoids (ECs), Lipogenesis, Nonalcoholic fatty liver disease (NAFLD), Receptor cannabinoid (CB1,CB2)

## Abstract

**Background:**

The endocannabinoids system (ECs) mediated mainly by CB1 and CB2 receptors plays an important role in non-alcoholic fatty liver disease by regulating lipid metabolism. This study is to further investigate the expression of CB1 and CB2 in the fat accumulation liver cells and to identify possible underlying mechanism by detecting the key lipogenesis factors.

**Methods:**

Sodium oleate and sodium palmitate were added into the HepG2 cell line for forming fat accumulation liver cell. MTT assay was used to test the cell’s cytotoxicity. The accumulation rate of fat in HepG2 cell was analyzed by the fluorescent staining. The mRNA and protein expression levels of CB1, CB2, SREBP-1c, ChREBP, L-PK, ACC1, FAS, LXRs and RXR were detected by RT-PCR and Western blot before and after the use of the antagonist.

**Results:**

The receptors of CB1 were expressed in HepG2 cells with low levels while in HepG2 fatty liver cells with higher levels (*p < 0.05*). However, after the application of antagonist, the expressions were significantly decreased (*p < 0.05*). The expressions of SREBP-1c, ChREBP and LXRs were detectable in HepG2 cells and the expressions were increased in HepG2 fatty liver cells (*p < 0.05*). After using the antagonists, the expressions of SREBP-1c, ChREBP, LXRs, ACC1 and FAS were significantly decreased (*p < 0.05*). But L-PK and RXR changed little in two groups (*p* > 0.05).

**Conclusion:**

Results of the present study demonstrated that CB1 receptors had important pathophysiological effects on the formation of fatty liver. CB1 receptors could be regulated by SREBP-1c, ChREBP and LXRs. Therefore, targeting CB1 receptors for the treatment of NAFLD might have a potential application value.

## Introduction

Non*-*alcoholic fatty liver disease (NAFLD) is a spectrum ranging from pure fatty liver to the more severe steatohepatitis, a condition that may progress to cirrhosis and even hepatocellular carcinoma [1]. Lipogenesis plays a critical role in the progression of NAFLD [2]. Though the potential mechanisms of lipogenesis in NAFLD are discussed a lot in previous studies, the etiology remains elusive [3].

Recently, it is reported that the endocannabinoids system (ECs) plays an important role in NAFLD and even its complications such as cardiovascular diseases are through modulating lipid metabolism [4]. ECs is primarily comprised of three components: endocannabinoids, endocannabinoid receptors, and endocannabinoid-metabolizing enzymes. Endocannabinoids, including arachidonoyl ethanolamide (anandamide) and 2-arachidonoylglycerol (2-AG), are lipid mediators that interact with cannabinoid receptors to produce effects similar to those of delta 9-tetrahydrocannabinol(THC), which is the main psychoactive component of cannabis. It has been reported that ECs widely participates in central and peripheral lipid metabolism through activating G protein-coupled cannabinoid receptors type 1 and type 2 (CB1 and CB2) [5]. ECs can not only stimulate the appetite to increase energy intake through the central nervous system, but also promote lipogenesis of peripheral tissues such as the adipose tissue, liver and skeletal muscle, thus leading to obesity and fatty liver disease [6, 7]. High fat diet, alcohol and endotoxin can stimulate the production of ECs [8]. Considering that CB1 receptors are distributed in the brain, adipose tissue, pancreas, gastrointestinal tract, skeletal muscle, heart and reproductive system, while CB2 receptors are mainly expressed in the immune system [9–11], it’s interesting to explore how CB1 and CB2 receptors involved in endocannabinoids to induce obesity and fatty liver.

More and more evidences show that high-fat diet-induced steatosis resulting from increased fatty acid synthesis is mediated via anandamide-induced CB1 receptor activation. Activation of CB1 receptors can increase hepatic gene expression of the lipogenic transcription factor SREBP-1 and its target enzymes, acetylCoA carboxylase-1(ACC1) and FAS. These effects are blocked or prevented by CB1 antagonist. CB1 receptor activation also appears to regulate fatty acid oxidation by modulating the activity of hepatic carnitine palmitoyltransferase1(CPT-1), the rate-limiting enzyme in fatty acid β-oxidation [12].

In past decades, some major lipogenesis-controlling factors have been identified in fatty liver disease, such as sterol regulatory element binding protein (SREBP1c), carbohydrate responsive elements binding protein (ChREBP) and liver X receptors (LXRs) [13]. Induced by insulin, SREBP1c regulates some of the key enzymes in fatty acid synthesis such as acetyl-CoA carboxylase (ACC1), fatty acid synthase (FAS). But studies find out that SREBP1c can only controlled 50% of fatty acid synthesis *in vivo* [14] *.* ChREBP can act on lipogenic gene promoters and regulate glucose to go into lipid synthesis pathway through the key enzymes liver pyruvate kinase (L-PK) [15]. As the oxysterols-activated nuclear receptors, LXRs are involved in cholesterol metabolism and also can induce liver lipogenesis. They act with the retinoid X receptors (RXRs) forming heterodimers to induce the expression of ACC, FAS and Stearoyl coenzyme A desaturase 1(SCD1). Moreover, LXRs can directly modulate the transcription of SREBP1c and ChREBP [16].

This study attempted to explore the possible mechanism underlying lipogenesis in the fat accumulation liver cells through investigating the expression of CB1 and CB2 receptors as well as SREBP1c, ChREBP, LXRs and the downstream factors ACC1, FAS, L-PK and RXRs.

## Methods

### Cell line and cell culture

HepG2 cells were seeded (1 × 10^7^ cells/100-mm dish) and cultured in RPMI1640 (Life Technology, INc., Grand Island, NY) containing 10% fetal bovine serum (Life Technology, INc.) for 24 h growing as adherent cell. All cell lines were maintained at 37°C in a humidified incubator with an atmosphere of 5% CO_2_.

### Methyl Thiazolyl Tetrazolium (MTT) assay

The cytotoxicity of the cells was measured by MTT assay. Stock solutions of fatty acids (10% w/v) prepared in serum-free RPMI1640 containing 1% BSA were conveniently diluted in culture medium to obtain the desired final concentrations. Sodium oleate and sodium palmitate (Sigma Aldrich, St. Louis, MO, USA) were added into the cultured cells for 24 hr at ratios of 3:0, 2:1, 1:1, 1:2 and 0:3, respectively. Sodium oleate and sodium palmitate were mixed at the concentrations of 1.5 mmol/L, 1.0 mmol/L, 0.75 mmol/L, 0.5 mmol/L and 0.25 mmol/L. CB1 receptor antagonist, rimonabant, was added to the cultured HepG2 fatty cells at the concentrations of 1 mmol/L, 5 mmol/L, 10 mmol/L, 20 mmol/L and 40 mmol/L for 4 hr, 8 hr, 12 hr, 24 hr and 48 hr, respectively.

### Fluorescence microscopy assay for fat accumulation liver cells

Stock solutions of nile red (Sigma Aldrich, St. Louis, MO, USA, 1000 ug/ml) in acetone were prepared and stored protected from light. The dye was added directly to the preparation to effect a 1:100 dilution. The specimen was incubated for 5 min. PBS rinsed the specimen while we removed excess dye. The accumulation rate of fat in HepG2 cell was analyzed by the fluorescent microscopy (excitation at 488 nm, and emission at 550 nm).

### Reverse transcription-polymerase chain reaction (RT-PCR)

The mRNA expression under different experimental conditions was assessed by RT-PCR. Total RNA was extracted using Trizol reagents (Invitrogen, USA) and the RT-PCR kit was used according to the manufacturer’s instructions (Shanghai Sangon Biotech Co., Ltd). The resulting single-stranded cDNA was denatured at 95°C for 3 min, and after the addition of the polymerase, subjected to 30 cycles of amplification, each consisting of 45 sec at 94°C, 45 sec at 57°C, and 45 sec at 72°C, with a 10- min final extension at 72°C during the last cycle. The primer sequences for CB1, CB2, SREBP-1c, ChREBP, LXRs, L-PK, ACC-l, FAS, RXRs and β-actin were described in Table 1. The PCR products were resolved by electrophoresis on 1.2% agarose gel and visualized with 0.5% ethium bromide.

**Table 1:** **The primers sequences of related genes**

Gene		Forward primer	
	5’	Reverse primer	3’
GAPDH		TGCACCACCAACTGCTTAGC	
		GGCATGGACTGTGGTCATGAG	
CB1		CTCGGACATTTTCCCACTC	
		AGGCAAACACCBTCTTGATA	
CB2		CCTCGTACCTGTTCATCG	
		TGTCCTGGTGCTACGTCAA	
SREBP-1C		GCG CTG CAG GCTGTA GGA TG	
		CTG CAC GGC TGT GCCAGG AG	
ChREBP		CCC TCA GAC ACC CAC ATC TT	
		CAG AGC TCA GAA AGG GGT TG	
LXRa		AGCGTCCACTCAGAGCAAGT	
		GGGGACAGAACAGTCATTCG	
L-PK		GAACACCTCTGCCTTCTGGA	
		CCCTGCACAAATCTCACAAA	
Acc-1		ACAGTGGAGCTAGAATTGGAC	
		ACTTCCCGACCAAGGACTTG	
Fas		AGGGGTCGACCTGGTCCTCA	
		GCCATGCCCAGAGGGTGGTT	
RXRa		GCACGTACACCGGAACA	
		CGCTTCTAGTGACGCATA	

### Western blot analysis

Cells were lysed with lysis buffer [50 mM Tris–HCl(pH 7.5), 250 mM NaCL, 0.1% NP40, 5 mM EGTA containing 50 mM sodium fluoride, 60 mM β-glycerol-phosphate, 0.5 mM sodium vanadate, 0.1 mM phenylmethylsulfonyl fluoride, 10 ug/ml aprotinin, and 10 ug/ml leupeptin]. Protein concentration was detected with BCA Protein Assay Reagent Kit (Pierce, Rockford, IL). Protein samples were electrophoresed in a 10% denaturing SDS gel and transferred to PVDF membrane (Bio-Rad, Califonia, US). The blots were incubated with specific primary antibodies, reacted with a peroxidase–conjugated secondary antibody (Santa Cruz Biotechnology, santa Cruz, CA), and finally visualized by enhanced chemiluminescence (Amersham, Piscataway, NJ). Polyclonal antibodies recognizing CB1/CB2, SREBP-1c, ChREBP, FAS, LXRs and RXR were purchased from Santa Cruz Biotechnology; L-PK, ACC1 monoclonal antibodies were purchased from Sigma-Aldrich co.

### Statistical analysis

Data were expressed as the means of at least three different experiments (mean ± SEM). The results were analyzed by Student’s test or one-way analysis of variance (SPSS18). And *p < 0.05* was considered statistically significant.

## Results

### Establishment of HepG2 fatty liver cells

**Figure 1:**
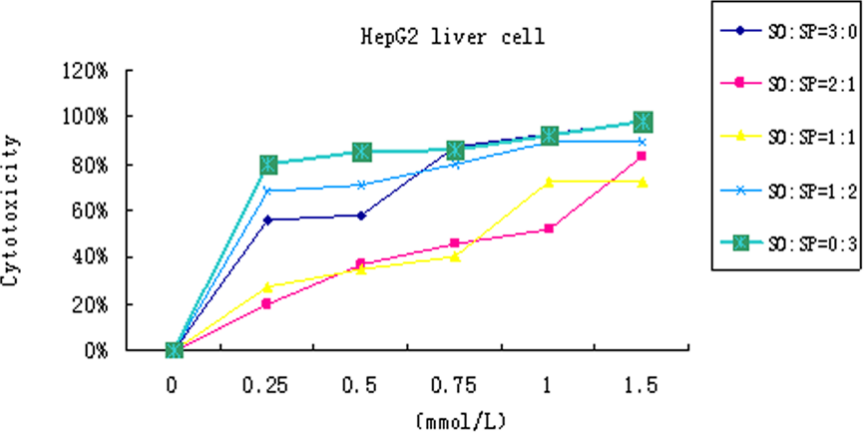
**MTT of mixed concentration of sodium oleate and sodium plamitate.**

**Figure 2:**
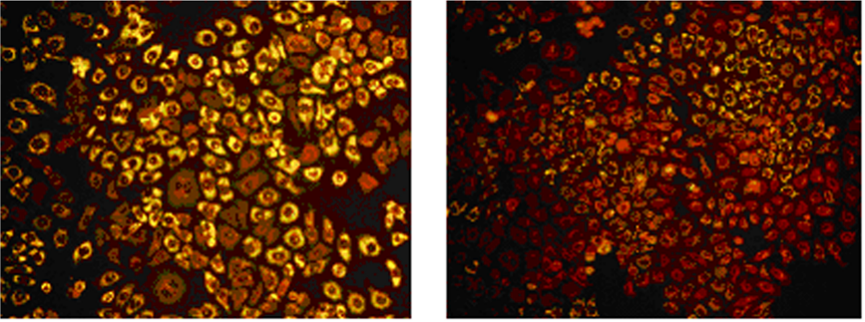
**Fat accumulation in HepG2 liver cell.** Fat accumulation rate in the HepG2 fatty liver cells was 52.1 ± 5.2%, while the rate in control group was 15.3 ± 6.6% (*p < 0.05*). HepG2 fatty liver cell; HepG2 liver cell.

**Figure 3:**
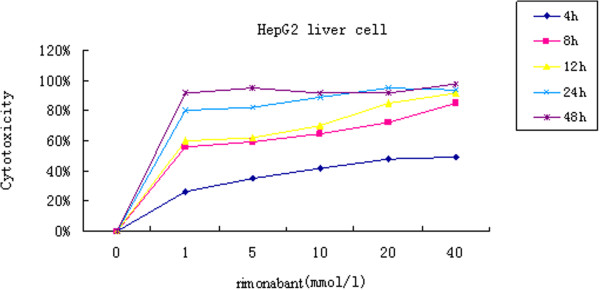
**MTT test of rimonabant.** CB1 receptor antagonist, rimonabant, was added to the cultured HepG2 fatty cells at the concentrations of 1 mmol/L, 5 mmol/L, 10 mmol/L, 20 mmol/L and 40 mmol/L for 4 hr, 8 hr, 12 hr, 24 hr and 48 hr, respectively.

Our MTT assays showed that the cell death was positively related to sodium palmitate concentration. According to the MTT test result, HepG2 cell added with sodium oleate and sodium palmitate at ratios of 2:1, 1:1 and 1:2 with the mix concentrations of 1.5 mmol/L, 1.0 mmol/L, 0.75 mmol/L and 0.5 mmol/L cultured for 24 hr was tested by the fluorescence assay, respectively. The best concentration with higher fat accumulation rate and higher cell’s viability was chosen by the image analysis system. The fluorescence assay was coincident with the MTT result. The mixed concentration of 1.0 mmol/L of sodium oleate and sodium palmitate at a ratio of 2:1 had minimal cell toxicity, higher cell viability and higher fat accumulation rate (Figure 1). It showed that the fat accumulation rate in the HepG2 fatty liver cells was 52.1 ± 5.2%, while the rate in control group was 15.3 ± 6.6% (*p < 0.05*) (Figure 2). MTT assay found that the cell’s viability was decreased with the increase concentration and the prolonged action period of rimonabant. The optimum concentration of rimonabant was 40 mmol/L and treating HepG2 cell for 4 hr (Figure 3)

.

### Expressions of CB1 and CB2 receptors in HepG2 fatty cells

**Figure 4:**
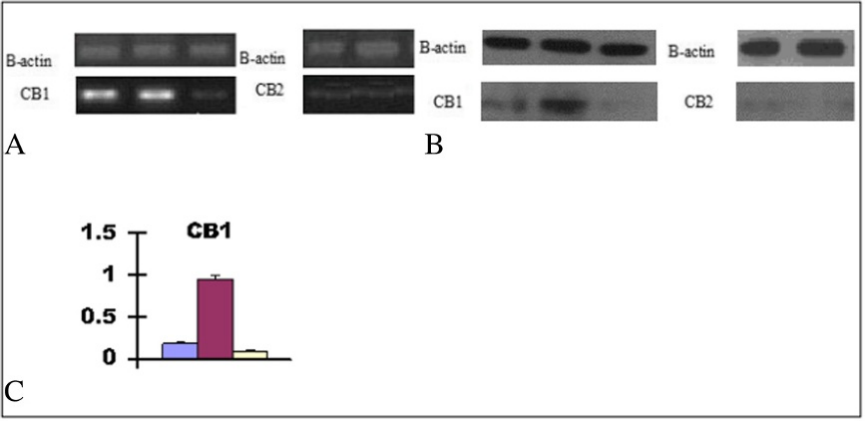
**The expressions of CB1 and CB2 in HepG2 liver cells. A**. The mRNA expression of CB1 and CB2 in HepG2 liver cells. **B**. The protein expression of CB1 and CB2 in HepG2 liver cells. **C**. The CB1 protein expression fold change in HepG2 cell, HepG2 fatty cell and rimonabant/HepG2-fatty cell was 0.28 ± 0.02,0.95 ± 0.04,0.12 ± 0.01 compare to B-actin as control, respectively (*p < 0.05*). HepG2; HepG2-fatty; Rimonabant/ HepG2-fatty. HepG2; HepG2-fatty. HepG2; HepG2-fatty; Rimonabant/HepG2-fatty.

The expression of CB1 receptor was increased in HepG2 fatty liver cells compare to HepG2 cells (*p < 0.05*). However, after application of the antagonist, rimonabant, the expression of CB1 receptor was significantly decreased (*p < 0.05*). The expressions of CB2 receptors in HepG2 cells and HepG2 fatty liver cells were undetectable (Figure 4A-4C).

### Expressions of SREBP-1c, ChREBP, L-PK, LXRs, ACC1, FAS and RXR in HepG2 fatty liver cells

**Figure 5:**
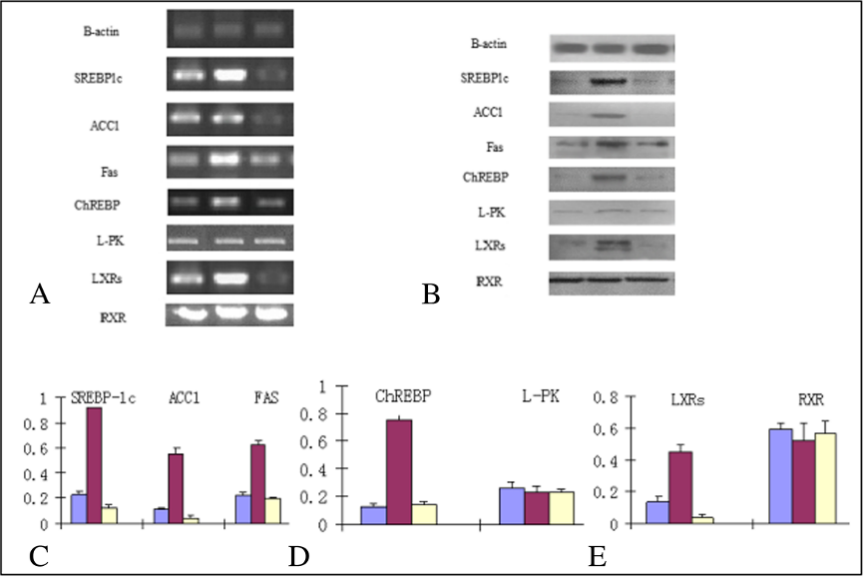
**The expressions of lipogenesis factors in HepG2 liver cells. A**. The mRNA expression of lipogenesis factors in HepG2 liver cells. **B**. The protein expression of lipogenesis factors in HepG2 liver Cells. **C-E**. The protein expression fold change. HepG2; HepG2-fatty; Rimonabant/HepG2-fatty. “Violet square symbol” HepG2 cell, “red square symbol” HepG2-fatty liver cell, “yellow square symbol” Rimonabant/HepG2-fatty liver cell.

The expression levels of SREBP-1c, ChREBP, L-PK, LXRs, ACC1, FAS and RXR were detected by RT-PCR and Western blot assay before and after the treatment of rimonabant to HepG2 fatty liver cells. SREBP-1c was expressed in HepG2 cells and the expression was increased in HepG2 fatty liver cells (*p < 0.05*). However, after use of the rimonabant, the expression of SREBP-1c was significantly decreased (*p < 0.05*). In the same way, the downstream factor ACC1 and FAS expressions were increased in HepG2 fatty liver cells compare to HepG2 cells, and after application of the antagonist, their expressions were decreased (*p < 0.05*). HepG2 cells showed little expression of ChREBP while the expression was significantly increased in HepG2 fatty liver cells (*p < 0.05*). After application of the antagonist, rimonabant, the expression of ChREBP was decreased (*p < 0.05*). Similarly, HepG2 cells showed little expression of LXRs while LXRs expression was significantly increased in HepG2 fatty liver cells (*p < 0.05*). After application of rimonabant, the expression of LXRs was decreased (*p < 0.05*). However, L-PK and RXR changed little in the two groups stated above, indicating that, in addition to CB1 and CB2 receptor-mediated regulation, L-PK and RXR could be regulated by other factors (*p* > 0.05) (Figure 5A-5E) (Table 2).

**Table 2:** **The protein expression of lipogenesis factors**

	HepG2 cell	HepG2 fatty cell	rimonabant/HepG2 fatty cell	F value	P value	
SREBP-1C	0. 2267 ± 0. 2517	0. 9200 ± 0. 2646	0. 1233 ± 0. 2517	858. 864	0. 000	p < 0.05
ACC1	0. 1100 ± 0. 0100	0. 5533 ± 0. 0503	0. 0433 ± 0. 0208	225. 533	0. 000	p < 0.05
ChREBP	0. 1233 ± 0. 0251	0. 7533 ± 0.0503	0. 1433 ± 0. 0208	320. 583	0. 000	p < 0.05
LPK	0. 2567 ± 0. 0450	0. 2267 ± 0. 0450	0. 2267 ± 0. 0288	0. 551	0. 000	p > 0.05
LXRs	0. 1367 ± 0. 0321	0. 4500 ± 0. 0500	0. 0400 ± 0. 0173	107. 852	0. 000	p < 0.05
RXR	0. 5933 ± 0. 0416	0. 5233 ± 0. 1069	0. 5700 ± 0. 0755	0. 606	0. 576	p > 0.05

## Discussion

In this study, we found the expressions of CB1 receptors were increased in HepG2 fatty liver cells compared to HepG2 cells, and also with the same results of the lipogenesis factors SREBP-1c, ChREBP and LXRs. While treated with the antagonist of CB1, the expressions of these lipogenesis factors including their downstream factors ACC1 and FAS were significantly decreased. However, there were no differences of L-PK and RXR expressions between two groups stated above.

HepG2 cell line was found to be suitable for investigating the impact of fat overaccumulation in the liver compare to normal human hepatocytes [17], and was also used for NAFLD research in many studies [18]. In this study, we added mixed fats of sodium oleate and sodium palmitate to HepG2 cell line as an *in vitro* non-alcoholic fatty liver disease model which included satuarated and unsaturated fatty acids.

Until now, many studies have reported that CB1 and CB2 receptors involved in endocannabinoids induced obesity and fatty liver. In mice model, expressions of CB1 and CB2 receptors were lower or none in normal liver, but increased significantly after high fat diet [19]. In the mice model with CB1 and CB2 receptors activating, obesity and fatty liver were formed progressively but not in rimonabant-treated mice or CB1 receptor knockout mice [20, 21]. Our *in vitro* study revealed that the expression of CB1 receptors was significantly increased in HepG2 fatty liver cells compared to HepG2 cells, and decreased after treated with antagonist, rimonabant. However, the expression of CB2 receptors remained unchanged in two groups. Therefore, we speculated that CB1 receptors played an important role in the lipogenesis of NAFLD and inhibiting CB1 receptors might improve lipogenesis in NAFLD.

Activation of CB1 receptor could induce the expression of transcription factor SREBP-1c and its downstream key enzymes such as ACC1 and FAS to enhance lipogenesis in vivo [22, 23]. By using HepG2 fatty liver cells, we also found that the expressions of SREBP-1c and its downstream factors ACC1 and FAS were increased in the progression of lipogenesis, but significantly decreased after treated with CB1 receptor antagonist. Furthermore, Our study *firstly* found that the expressions of ChREBP and LXRs were significantly increased in HepG2 fatty liver cells but decreased after treated with the antagonist. However, there was no obvious expression changing of downstream factors L-PK and RXR. According to these findings, we got the conclusion that inhibiting CB1 receptors could decrease the expressions of lipogenesis factors, SREBP-1c, ChREBP and LXRs, thus improve lipogenesis in non-alcoholic fatty liver disease.

Andrea De Gottardi, et al. reported that cannabinoid receptors were downregulated in the presence of steatosis.SREBP-1c and FAS were downregulated in fatty immortalized human hepatocytes. These results were different from our study [24]. The probable reasons might be that in their study they used oleic acid to induce immortalized human hepatocytes steatosis for 7 days, while we used sodium oleate and sodium palmitate to induce HepG2 cell steatosis for 24 h. We speculated that saturated and unsaturated fatty acids might have different effects on hepatocytes and these might need further to study.

In conclusion, CB1 receptor participated in ECs and induced lipogenesis in hepatocytes through factors SREBP-1c, ChREBP and LXRs, and might be the target for the treatment of NAFLD [25].
